# Molecular mechanism of DNA damage induced by titanium dioxide nanoparticles in toll-like receptor 3 or 4 expressing human hepatocarcinoma cell lines

**DOI:** 10.1186/s12951-014-0048-2

**Published:** 2014-12-02

**Authors:** Karim Samy El-Said, Ehab Mostafa Ali, Koki Kanehira, Akiyoshi Taniguchi

**Affiliations:** Cell-Material Interaction Group, Biomaterial Unit, Nano-Bio Field, Interaction Center for Material Nanoarchitectonics (MANA), National Institute for Materials Science (NIMS), Tsukuba, Japan; Graduate School of Advanced Science and Engineering, Waseda University, Shinjuku, Japan; Department of Chemistry, Faculty of Science, Tanta University, Tanta, Egypt; Biotechnology Group, TOTO Ltd. Research Institute, Honson 2-8-1, Chigasaki, Kanagawa 253-8577 Japan; National Institute for Materials Science (NIMS), 1-1 Namiki, Tsukuba, Ibaraki 305-0044 Japan

**Keywords:** *TiO*_*2*_*NPs*, *TLRs*, *DNA damage*, *ROS*, *Apoptosis*

## Abstract

**Background:**

Titanium dioxide nanoparticles (TiO_2_ NPs) are widely used in the biological sciences. The increasing use of TiO_2_ NPs increases the risk of humans and the environment being exposed to NPs. We previously showed that toll-like receptors (TLRs) play an important role in the interactions between NPs and cells. Our previous results indicated that TLR4 increased the DNA damage response induced by TiO_2_ NPs, due to enhanced NP uptake into the cytoplasm, whereas TLR3 expression decreased the DNA damage response induced by TiO_2_ NPs because of NP retention in the endosome. In this study, we explored the molecular mechanism of the DNA damage response induced by TiO_2_ NPs using TLR3 or TLR4 transfected cells. We examined the effect of TLR3 or TLR4 over-expression on oxidative stress and the effect of DNA damage induced by TiO_2_ NPs on gene expression levels.

**Results:**

Our results showed evidence for elevated oxidative stress, including the generation of reactive oxygen species (ROS), with increased hydrogen peroxide levels, decreased glutathione peroxidase, and reduced glutathione and activated caspase-3 levels in cells exposed for 48 h to 10 μg/ml TiO_2_ NPs. These effects were enhanced by TLR4 and reduced by TLR3 over-expression. Seventeen genes related to DNA double-strand breaks and apoptosis were induced, particularly *IP6K3* and *ATM*.

**Conclusion:**

Our results indicated that TiO_2_ NPs induced ROS, and the above molecules are implicated in the genotoxicity induced by TiO_2_ NPs.

## Background

Nanotechnology is one of the fastest growing sectors of the high-tech economy. Several consumer products currently use nano-materials; these products have personal, commercial, medical, and military uses [1,2]. Engineered nano-materials are suited to a wide range of novel applications in the electronics, healthcare, cosmetics, technologies and engineering industries. The dearth of toxicological data on nano-materials makes it difficult to determine if there is a risk associated with exposure to nano-materials. Thus, there is an urgent need to develop rapid, accurate and efficient testing strategies to assess the health effects of these emerging materials [3].

Titanium dioxide nanoparticles (TiO_2_ NPs) possess dramatically different physicochemical properties compared to TiO_2_ fine particles (FPs). TiO_2_ NPs are widely used in the biomedical and bioengineering fields due to their strong oxidizing properties and chemical inertness [4]. Moreover, TiO_2_ nano-materials are widely used in industrial and consumer products due to their strong catalytic activity attributed to their small size, which provides a larger surface area per unit mass. These properties of TiO_2_ NPs may present both unique bioactivity properties and challenges to human health [5-7]. Indeed, the physicochemical properties of TiO_2_ NPs have been demonstrated to correlate with their toxicological effects [8]. TiO_2_ nano-materials have attracted much interest in medical fields due to their photo-reactivity [9]. It was previously reported that a TiO_2_ photo-catalyst can kill bacterial cells in water due to the generation of compounds such as reactive oxygen species (ROS) [10,11]. Furthermore, photo-excited anatase TiO_2_ nanoparticles effectively induce cytotoxicity in HeLa cancer cells [12]. TiO_2_ NPs are used increasingly in industrial products, such as toothpastes, sunscreens, cosmetics, pharmaceuticals, and food products [13]. Human exposure may occur during both the manufacturing process and use. The widespread use of TiO_2_ NPs, and their potential entry into the body through dermal, ingestion, and inhalation routes, suggests that TiO_2_ NPs pose a potential exposure risk to humans, livestock, and the ecosystem [14-18]. TiO_2_ NP toxicity may be due to the ease with which these NPs can pass through the cellular membranes and disrupt biological systems [19]. It has been suggested that the small size and corresponding large specific surface area are the major determinants of NP toxicity [20]. It has also been proposed that the large surface area of NPs greatly increases their ability to produce potentially toxic ROS [21].

ROS are reactive molecules and free radicals derived from molecular oxygen. These molecules are produced as byproducts during the mitochondrial electron transport of aerobic respiration or by oxidoreductase enzymes and metal catalyzed oxidation, and have the potential to cause a number of deleterious events. ROS play a role in cellular signaling, including apoptosis, gene expression, and the activation of cell signaling cascades [22]. Oxidative DNA damage induced by ROS and free radicals is important in the pathogenesis of many human diseases, including cancer, muscle degeneration, coronary heart disease and ageing [23]. Moreover, studies have indicated that TiO_2_ NPs induce photo-damage to DNA in human cells, mouse lymphoma cells, and phage [24-26].

Toll-like receptors (TLRs) play an essential role in the activation of innate immunity by recognizing specific molecular patterns of microbial components. TLRs are transmembrane proteins composed of both an extra-cellular domain (responsible for ligand recognition) and a cytoplasmic domain (required for initiating signaling) [27]. As suggested by their range of ligands and subcellular locations, TLRs recognize a wide range of foreign materials [28,29]. For example, TLRs that localize to the cell surface, such as TLR4, primarily recognize bacterial components. In contrast, TLRs that localize to the endocytic compartments, such as TLR3, mainly recognize viruses. We have previously shown that TLRs are also involved in the cellular response and cellular uptake of TiO_2_ NPs [30,31]. We have also shown that the exposure of HepG2 cells to TiO_2_ NPs induces DNA damage responses; this damage was increased by TLR4 over-expression, and decreased by TLR3 over-expression [32]. TLR3, which has a subcellular location distinct from TLR4, reduced the DNA damage response caused by TiO_2_ NPs. These results suggested that TLRs could be involved in many cellular responses, including genotoxicology. However, the molecular mechanism of DNA damage induced by TiO_2_ NPs is unknown.

In the present study, we aimed to elucidate the molecular mechanism of DNA damage induced by TiO_2_ NPs by using PCR array and real**-**time PCR (RT-PCR). Specifically, we examined the effect of TiO_2_ NP exposure on gene expression changes in DNA damage signaling pathways involving apoptosis, cell-cycle arrest, and DNA repair. Furthermore, we also confirmed elevated ROS generation by demonstrating increased hydrogen peroxide (H_2_O_2_) levels, decreased glutathione peroxidase (GPX) and glutathione (GSH) levels, as well as caspase-3 activation, in cells exposed to TiO_2_ NPs with or without TLR3 and TLR4 over-expression. We expect our work to advance the understanding of the molecular mechanism of DNA damage induced by TiO_2_ NPs.

## Results

**Figure 1:**
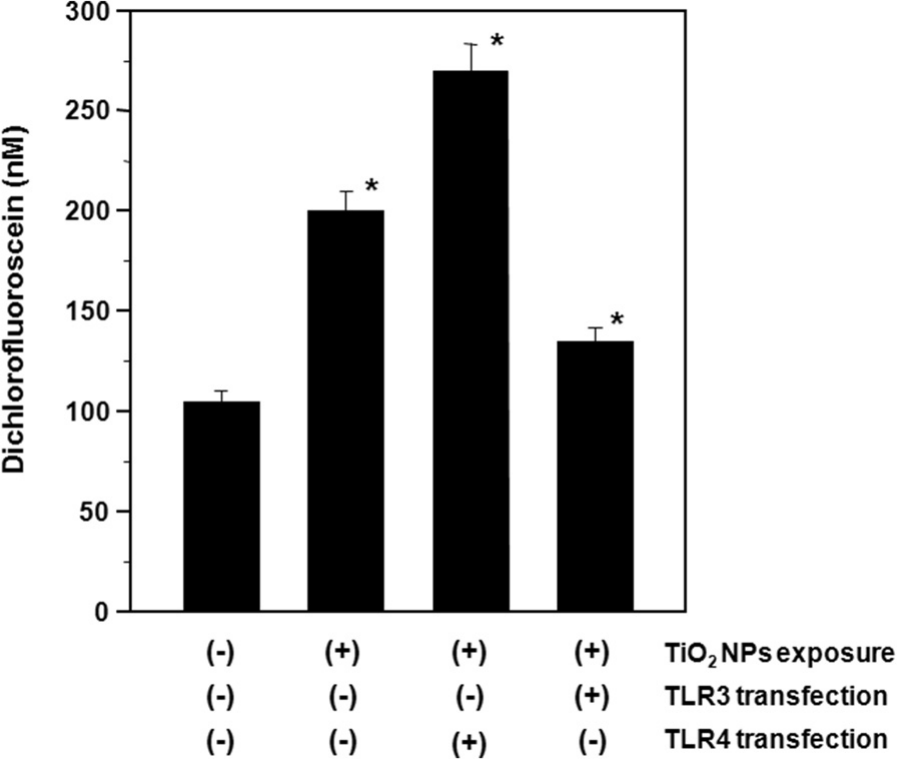
**Reactive oxygen species (ROS) generation in TiO**_**2**_ **NP-exposed HepG2 cells with and without TLR3 or TLR4 transfection.** The transfected cells were exposed to 10 μg/ml TiO_2_ NPs for 48 h. Each plot was produced from at least 3 replicate measurements. All values are presented as mean ± S.D. (n ≥3), (*P <0.05).

In general, ROS generation results in DNA damage. Our aim was to examine TiO_2_ NP-induced ROS generation and its association with DNA damage responses. ROS generation was measured in HepG2 cells exposed to TiO_2_ NPs with and without TLR3 or TLR4 over-expression (Figure 1). The results showed that in TiO_2_ NP-exposed cells, ROS levels were significantly increased (approximately 1.9 fold) compared with control cells (untreated, untransfected HepG2 cells). Cells exposed to TiO_2_ NPs and over-expressing TLR4 showed a significant increase in ROS levels compared to untransfected cells exposed to TiO_2_ NPs, reaching an approximately 2.6 fold increase compared to control. In comparison, ROS levels in HepG2 cells exposed to TiO_2_ NPs with TLR3 over-expression were slightly (≈1.3 fold) increased compared to control cells, as shown in Figure 1. These results indicate that ROS generation is a factor in the DNA damage response induced by TiO_2_ NPs.

**Figure 2:**
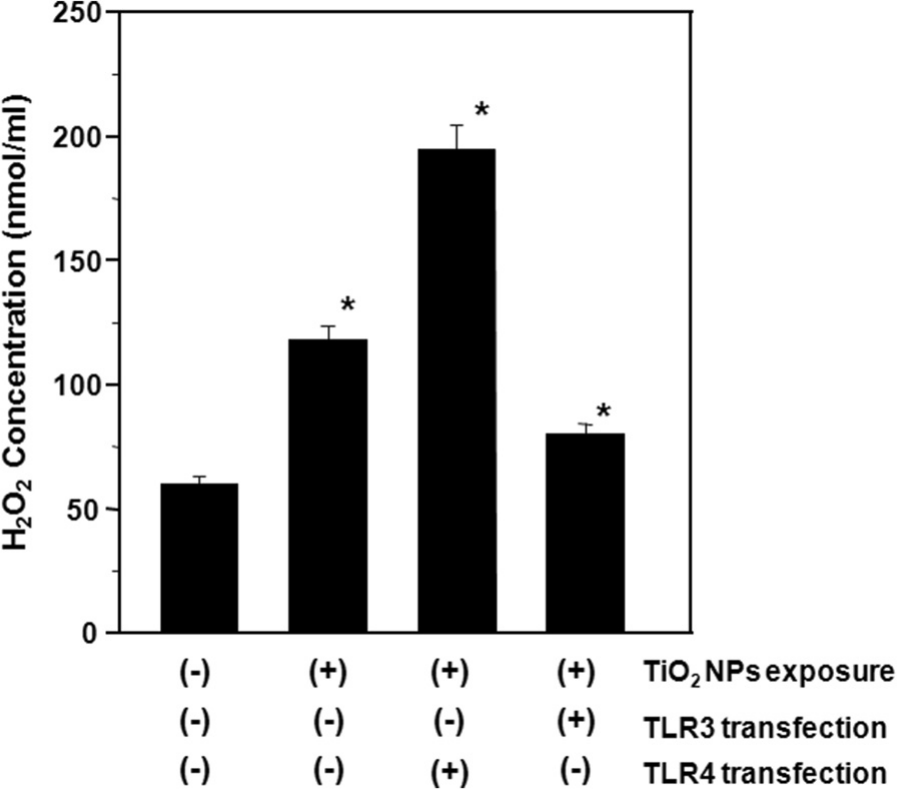
**Hydrogen peroxide (H**_**2**_ **O**_**2**_ **) levels in TiO**_**2**_ **NP-exposed HepG2 cells with and without TLR3 or TLR4 transfection.** The transfected cells were exposed to 10 μg/ml TiO_2_ NPs for 48 h. Each plot was produced from at least 3 replicate measurements. All values are presented as mean ± S.D. (n ≥3), (*P <0.05).

Oxidative stress reflects an imbalance between the systemic manifestation of reactive oxygen species and a biological system’s ability to readily detoxify the reactive intermediates. Certain oxidant and antioxidant parameters were evaluated in order to obtain information on the cellular mechanism of oxidative stress in response to TiO_2_ NP exposure, as well as the effect of TLR3 or TLR4 over-expression. H_2_O_2_ is a reactive oxygen metabolic byproduct that serves as a key regulator of a number of oxidative stress-related states. Measurement of this reactive species provides an indication of the modulation of intracellular pathways by oxidative stress during TiO_2_ NP exposure and TLR3 and TLR4 over-production. In the present study, H_2_O_2_ concentrations were elevated 1.9 fold in cells exposed to TiO_2_ NPs compared to control cells. H_2_O_2_ concentration was further increased in cells expressing TLR4 (3.2 fold), whereas cells expressing TLR3 exhibited only a 1.4 fold increase (Figure 2). Our data show that H_2_O_2_ is an intermediate in TiO_2_ NP-induced oxidative stress, regardless of TLR3 or TLR4 over-expression.

**Figure 3:**
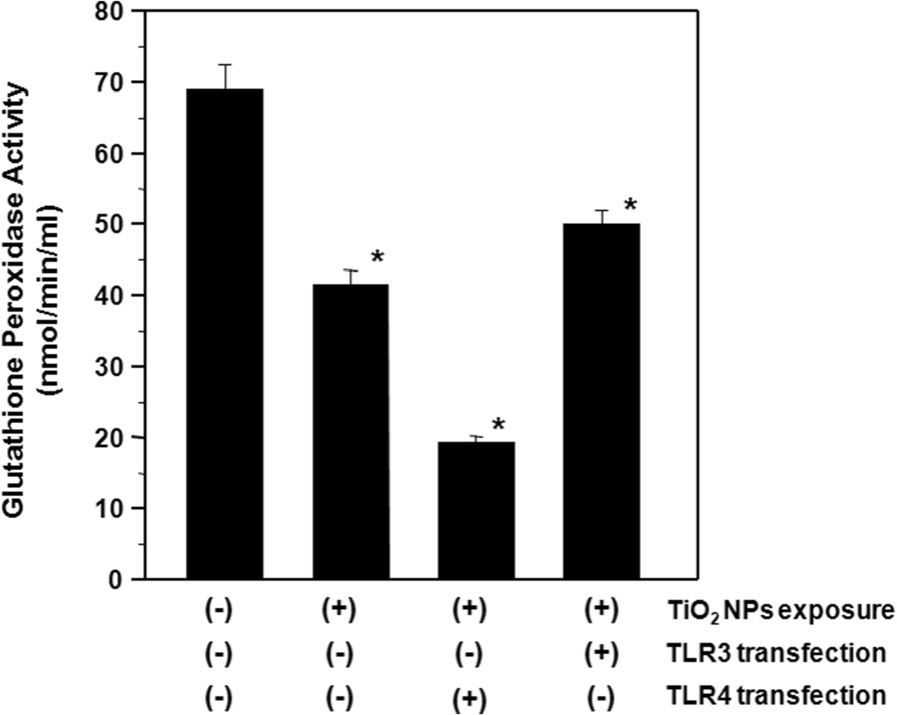
**Glutathione peroxidase (GPx) activities in TiO**_**2**_ **NP-exposed HepG2 cells with and without TLR3 or TLR4 transfection.** The transfected cells were exposed to 10 μg/ml TiO_2_ NPs for 48 h. Each plot was produced from at least 3 replicate measurements. All values are presented as mean ± S.D. (n ≥3), (*P <0.05).

**Figure 4:**
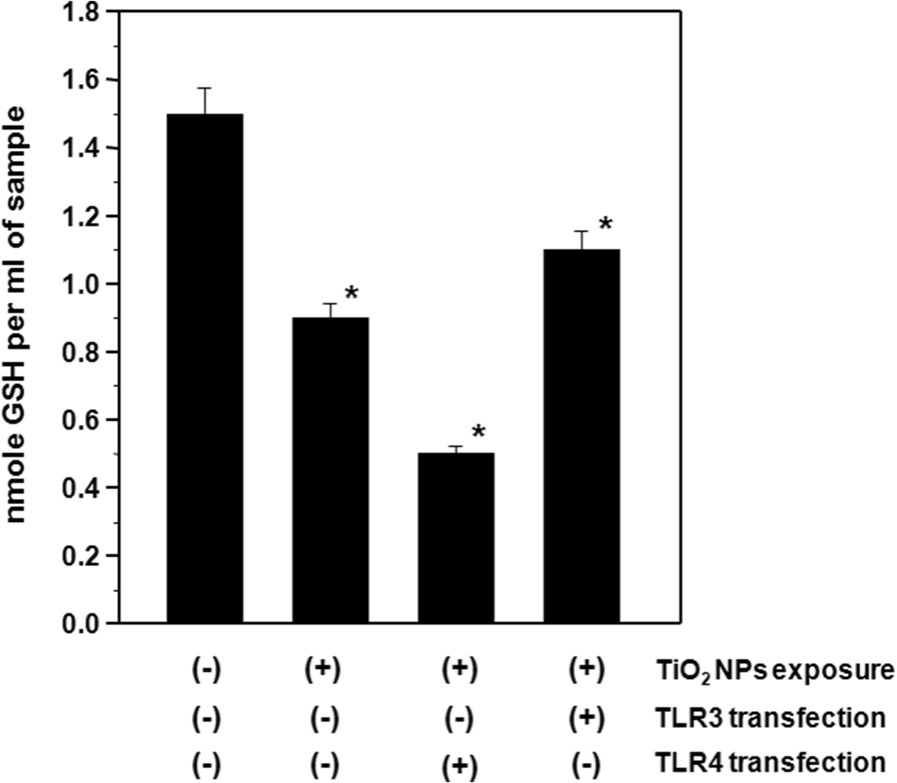
**Reduced glutathione (GSH) levels in TiO**_**2**_ **NP-exposed HepG2 cells with and without TLR3 or TLR4 transfection.** The transfected cells were exposed to 10 μg/ml TiO_2_ NPs for 48 h. Each plot was produced from at least 3 replicate measurements. All values are presented as mean ± S.D. (n ≥3), (*P <0.05).

GPX catalyzes the reduction of hydroperoxides, including H_2_O_2_, using GSH tripeptide as a hydrogen donor. In order to confirm the elevated H_2_O_2_ levels, we measured GPX activity and GSH levels. The results showed that TiO_2_ NP treatment inhibited GPX enzyme activity in HepG2 cells by 1.6 and 3.6 fold in the absence and presence of TLR4 transfection, respectively, when compared to control cells. Over-expression of TLR3 with TiO_2_ NP exposure resulted in a 1.3 fold decrease in GPX activity compared to control cells (Figure 3). Similarly, TLR4 over-expression exacerbated TiO_2_ NP-induced reductions in GSH levels, while TLR3 over-expression appeared to reduce the effects of TiO_2_ NP exposure (Figure 4). These results showed that reduced GPX detoxification of H_2_O_2_ is involved in the oxidative stress response stimulated by TiO_2_ NPs, regardless of TLR3 or TLR4 over-expression, and confirmed the accumulation of H_2_O_2_ due to inhibition of the antioxidants GPX and GSH.

**Figure 5:**
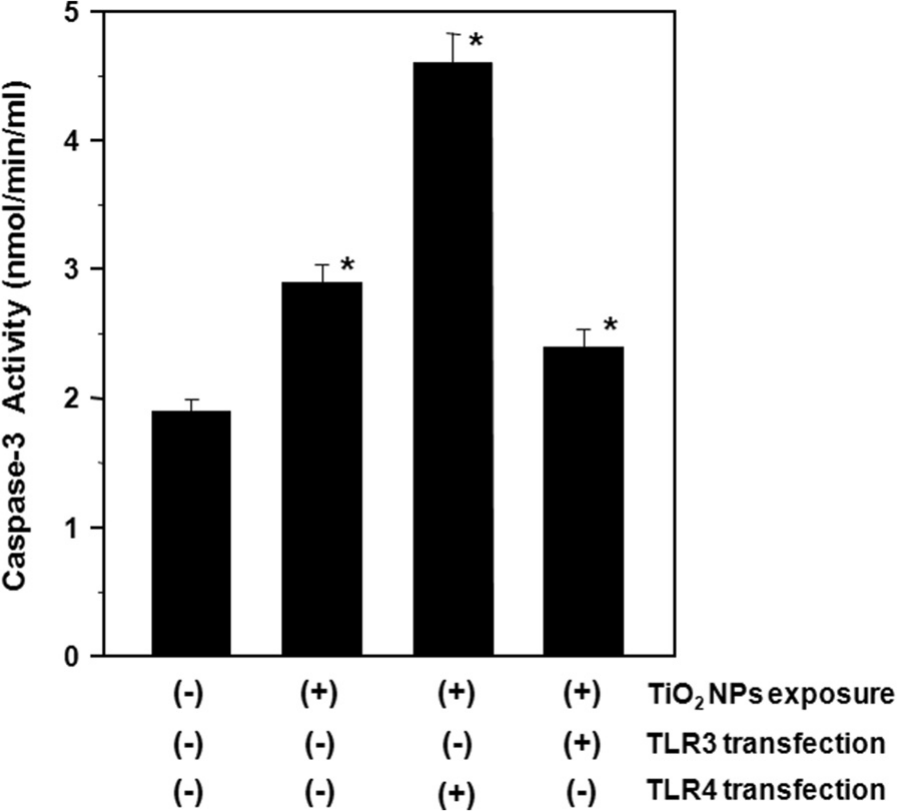
**Caspase-3 activities in TiO**_**2**_ **NP-exposed HepG2 cells with and without TLR3 or TLR4 transfection.** The transfected cells were exposed to 10 μg/ml TiO_2_ NPs for 48 h. Each plot was produced from at least 3 replicate measurements. All values are presented as mean ± S.D. (n ≥3), (*P <0.05).

Caspase-3, an effector cysteine protease involved in apoptosis and necrosis, is activated by H_2_O_2_ [33]. Therefore, monitoring caspase-3 activation is important for evaluating apoptotic responses to oxidative stress occurring in HepG2 cells exposed to TiO_2_ NPs. Our results showed that caspase-3 activity significantly increased in cells exposed to TiO_2_ NPs, and that TLR4 expression further increased caspase-3 activation. Conversely, TLR3 expression resulted in an almost complete reversal of TiO_2_ NP-induced caspase-3 activation (Figure 5). Therefore, TiO_2_ NP-treatment induces apoptosis involving elevated caspase-3 activity in TLR4-expressing and normal HepG2 cells.

**Figure 6:**
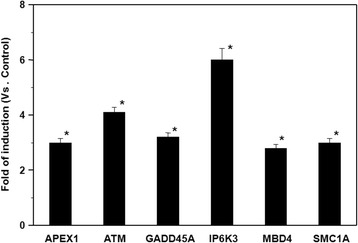
**Expression of DNA damage marker mRNAs in TiO**_**2**_ **NP-exposed HepG2 cells.** Cells were exposed to 10 μg/ml TiO_2_ NPs for 48 h. Results are shown as the mean ± SD, n ≥3 for each marker, (*P <0.05).

We used PCR array and RT-PCR to assess the cellular mechanisms operating in response to TiO_2_ NP exposure in the presence of TLR4 over-expression. The genes up-regulated by greater than 1-fold are listed in Table 1, and consist of genes in the human DNA damage signaling pathways (Table 1). In particular, the expression of the genes for apurinic/apyrimidinic exonuclease 1 (APEX1), ataxia telangiectasia mutated (ATM), growth arrest and DNA-damage-inducible, alpha (GADD45A), inositol hexakisphosphate kinase 3 (IP6K3), methyl-CpG binding domain protein 4 (MBD4), and structural maintenance of chromosomes 1A (SMC1A) were increased by >1.5 fold, with the remainder of the genes in the PCR array exhibiting <1.5 fold changes. In order to confirm the induction of the above-mentioned genes, the mRNA induction levels were determined by real-time PCR. The real-time PCR results confirmed the induction of mRNA expression observed for each of the genes (Figure 6). Indeed, the real-time PCR results indicated that the genes induced to the greatest extent were ATM and IP6K3, which is consistent with double-strand breaks in the DNA that result in DNA fragmentation and apoptosis [34].

**Table 1:** **Induction of gene mRNA expression in HepG2 cells transfected with TLR4 expression vectors**

**Symbols of genes**	**Description of the genes**	**Fold regulation**
ABL1	C-abl oncogen 1, non-receptor tyrosine kinase	1.56
**APEX1**	**APEX nuclease (multifunctional DNA repair enzyme) 1**	**1.86**
**ATM**	**Ataxia telangiectasia mutated**	**1.91**
ATR	Ataxia telangiectasia and Rad3 related	1.42
BRCA1	Breast cancer 1, early onset	1.23
CHEK2	CHK2 checkpoint homolog (S.pombe)	1.41
CIDEA	Cell death-inducing DFFA-like effector a	1.32
**GADD45A**	**Growth arrest and DNA-damage inducible, alpha**	**1.77**
GML	Glycosylphosphatidylinositol anchored molecule like protein	1.3
**IP6K3**	**Inositol hexaphosphate kinase 3**	**2.23**
MAPK6	Mitogen-activated protein kinase 6	1.34
**MBD4**	**Methyl-CpG binding domain protein 4**	**1.65**
BTG2	B-cell translocation gene 2	1.43
RAD18	RAD18 homolog (S. cerevisiae)	1.34
RAD21	RAD21 homolog (S. pombe)	1.39
**SMC1A**	**Structural maintenance of chromosomes 1A**	**1.62**
PNKP	Polynucleotide kinase 3′-phosphate	1.27

Cells exposed to 10 μg/ml TiO_2_ NPs for 48 h prior to gene expression analysis with PCR array. Fold regulation values greater than 1 indicate positive regulation (up-regulation).

**Figure 7:**
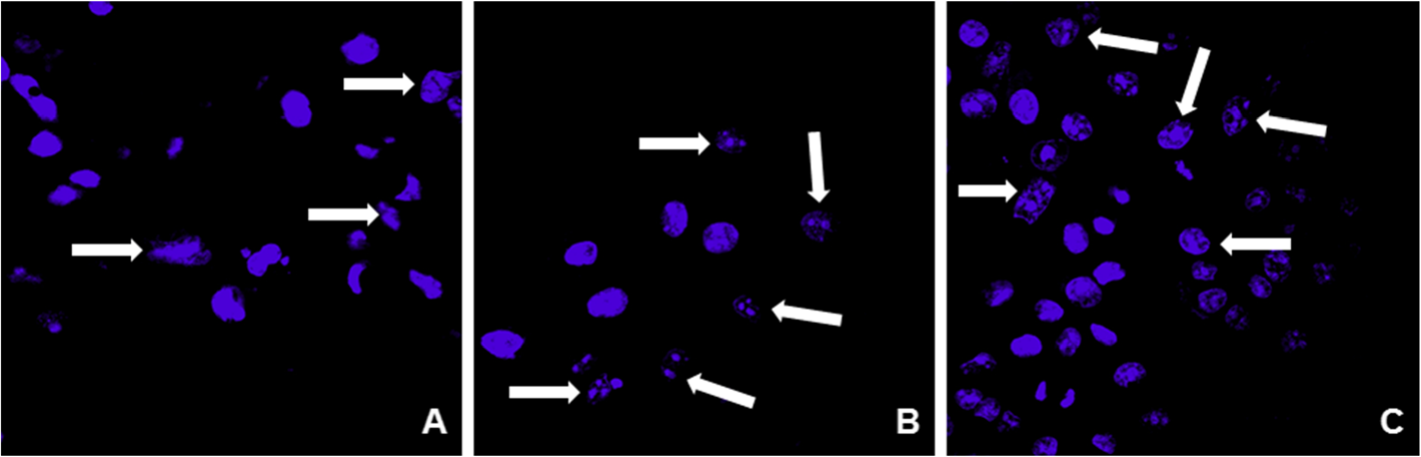
**Confocal laser scanning microscopic images of HepG2 cells, with and without TLR4 transfection, treated with TiO**_**2**_ **NPs. (A)** HepG2 cells without transfection and without TiO_2_ NP exposure, **(B)** Cells exposed to TiO_2_ NPs only, **(C)** Cells transfected with TLR4 expression vector and exposed to 10 μg/ml TiO_2_ NPs for 48 h. The white arrows show the apoptotic, nuclear fragmented cells. The confocal microscopic images show condensation of chromatin and nuclear fragmentation in HepG2 cells transfected with TLR4 expression vector and exposed to TiO_2_ NPs.

To confirm the enhancement of apoptosis in HepG2 cells, we used Hoechst DNA staining to observe nuclear fragmentation as an indication of apoptosis. As shown in Figure 7, morphological changes consistent with cellular apoptosis, including condensation of chromatin and nuclear fragmentation, were observed in the cells exposed to TiO_2_ NPs. Again, expression of TLR4 (Figure 7C) appeared to enhance the effects of TiO_2_ NPs (Figure 7B) on apoptosis. Microscopy analysis confirmed that cells exposed to TiO_2_ NPs and transfected with TLR4 undergo programmed cell death (apoptosis) because of DNA damage. HepG2 cells that did not express TLRs and that were not exposed to TiO_2_ NPs also underwent apoptosis: by counting the number of apoptotic cells, we determined that 28% of untreated, untransfected cells had fragmented nuclei (Figure 7A), 55% of HepG2 cells exposed to TiO_2_ NPs were apoptotic (Figure 7B), and 75% of cells over-expressing TLR4 and exposed to TiO_2_ NPs were apoptotic (Figure 7C).

## Discussion

**Figure 8:**
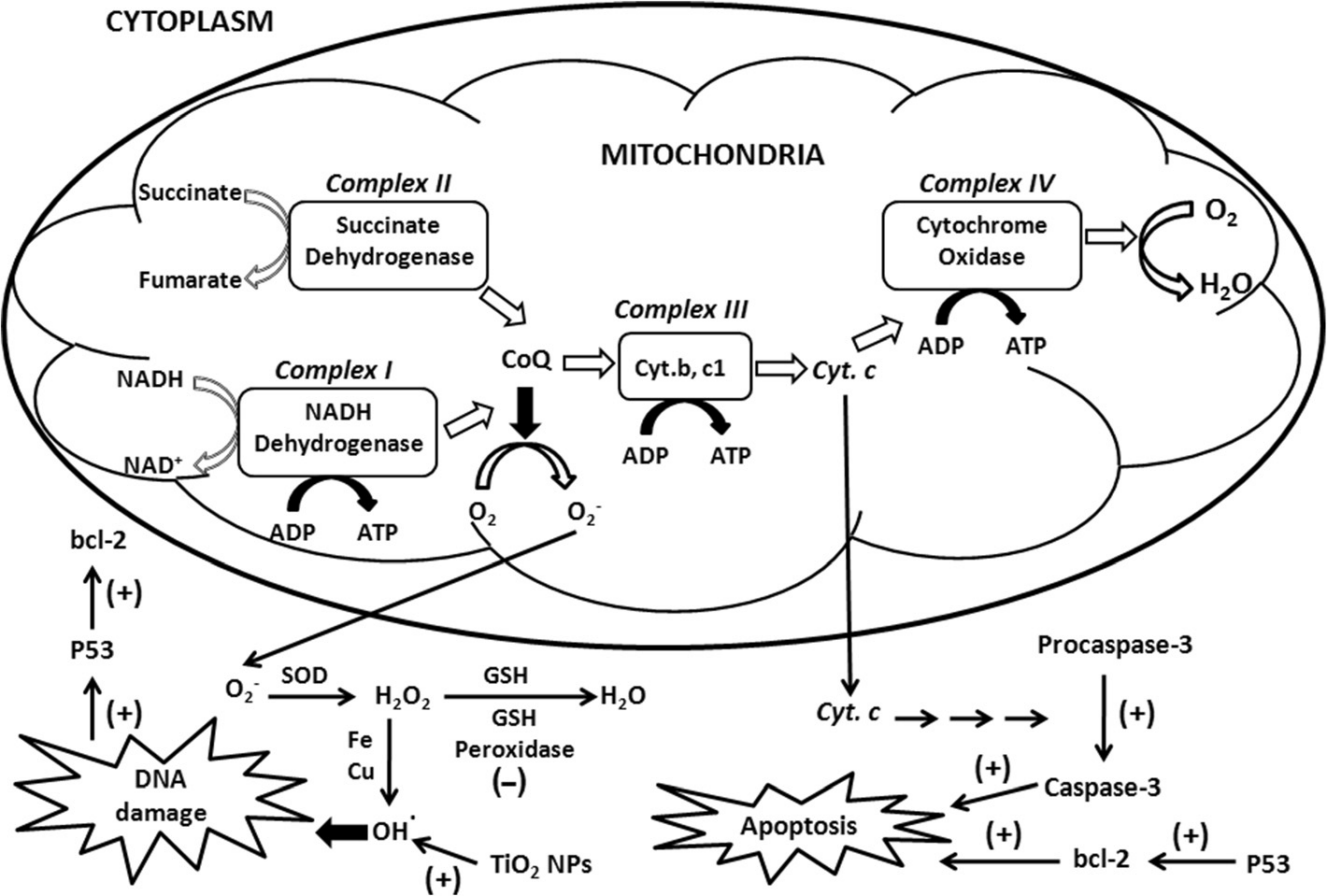
**A schematic representation of mitochondrial ROS implicated in DNA damage and apoptosis induced by TiO**_**2**_ **NP exposure, with and without TLR4 over-expression.** The figure shows the site of induction and inhibition of respiratory complexes and oxidative stress molecules involved in ROS generation in HepG2 cells exposed to TiO_2_ NPs with and without TLR4 over-expression. (+) indicate the sites of activation and (-) show the sites of inhibition.

The purpose of this study was to examine the molecular mechanism of DNA damage caused by exposure to TiO_2_ NPs (10 μg/ml). A high concentration of TiO_2_ NPs should amplify the effects of the NPs and thus aid examination of their mechanism of action. The interactions of NPs with cells resulted in the generation of ROS, and the resultant oxidative stress may cause DNA fragmentation [35,36]. We found a significant increase in ROS generation in cells exposed to TiO_2_ NPs, which is consistent with our previous report of DNA damage responses in TiO_2_ NP-exposed cells [32]. In this paper, the results indicated that TiO_2_ NPs induced oxidative stress in cells, which can cause oxidative DNA damage and lead to the activation of p53 tumor suppressors and bcl-2 apoptotic factors. Additionally, oxidative stress can affect the mitochondria, the richest source of ROS, in which oxygen is metabolized and converted to O_2_^−^ by several components of the mitochondrial respiratory chain (Figure 8).

TLRs recognize and respond to exogenous and endogenous ligands through signaling pathways, leading to inflammatory cascade mediator production, which directs the innate and adaptive immune responses. TLRs interact with microbial components, such as lipopeptides, and non-self nucleic acids [37]. TLR4 localizes to the cell surface and TLR3 localizes in the endosome. We have shown that TLR4 is involved in TiO_2_ NP-induced inflammatory responses and TiO_2_ NP-incorporation [38,39]. We also have shown that TLR3 and TLR4 are involved in DNA damage induced by TiO_2_ NPs, indicating that TLR3 reduces DNA damage while TLR4 exacerbates it [32]. In this paper, our results showed more significant effects in HepG2 cells exposed to TiO_2_ NPs with TLR4 over-expression due to increased TiO_2_ NP uptake into the cytoplasm and increased signal transduction involving ROS in TLR4-dependent activation of NF-kB [40], while TLR3 reduced the effects caused by TiO_2_ NPs.

Various endogenous and exogenous genotoxic insults induce DNA-damage checkpoint signaling. The biological outcomes of checkpoint signaling include the control and coordination of cell-cycle progression, transcription, DNA replication, DNA repair, and apoptosis. DNA lesions trigger the activation of various kinases, which constitute the primary transducers in the signaling cascade. Of utmost importance are the phosphoinositide-3-kinase-related protein kinase (PIKK) family members, ATM, ATR, and DNA-dependent protein kinase. While ATR activation is associated with single-stranded DNA and stalled DNA replication forks, ATM and DNA-dependent protein kinase respond mainly to DNA double-strand breaks (DSBs) [34]. To identify marker genes of DNA damage-related cytotoxic stimulation, PCR array and RT-PCR analysis were performed using a commercial array system. Our results showed the induction of six genes, as follows: (1) APEX1 gene: a multifunctional DNA repair enzyme that plays a central role in the cellular response to oxidative stress. The major function of APEX1 in DNA repair and redox regulation of transcriptional factors is as an apurinic/apyrimidinic (AP) endodeoxyribonuclease in the DNA base excision repair pathway of DNA lesions induced by oxidative and alkylating agents. (2) ATM genes: the protein encoded by this gene belongs to the PI3/PI4-kinase family. This protein is an important cell cycle checkpoint kinase that functions as a regulator of a wide variety of downstream proteins, including tumor suppressor protein p53. It also functions as a serine/threonine protein kinase that activates checkpoint signaling upon DSBs, apoptosis, and genotoxic stresses, thereby acting as a DNA damage sensor. (3) GADD45A gene: the protein encoded by this gene responds to environmental stresses by mediating activation of the p38/JNK pathway via MTK1/MEKK4 kinase. DNA damage-induced transcription of this gene is mediated by both p53-dependent and -independent mechanisms. (4) IP6K3 gene: encodes a protein of the IPK family. IP6Ks regulate numerous biological processes, including chemotaxis, telomere length, and apoptosis [41]. IP6K3 impacts cell death, induces p53-mediated apoptosis, and its over-expression sensitizes cells to diverse apoptotic stimuli. (5) MBD4 gene: the protein encoded by this gene belongs to a family of nuclear proteins related by the presence of a methyl-CpG binding domain (MBD). These proteins are capable of binding specifically to methylated DNA, and possess mismatch-specific DNA N-glycosylase activity involved in DNA repair. They can also remove uracil or 5-fluorouracil in G:U mismatches. (6) Finally, the sixth gene is SMC1A; the encoded protein is thought to be an important part of functional kinetochores. This protein interacts with BRCA1 and is phosphorylated by ATM, indicating a potential role for this protein in DNA repair. These data suggest that these six genes are useful markers for DNA damage signaling pathways in response to TiO_2_ NP exposure, with the highest induction observed with ATM and IP6K3, as illustrated in Figure 6. Given that ATM and IP6K3 gene induction are involved in DSBs [34], the type of DNA damage induced by TiO_2_ NP exposure is likely DNA DSBs that cause eventual DNA fragmentation and apoptosis.

## Conclusions

Our results showed that exposing HepG2 cells to TiO_2_ NPs enhances ROS generation and activates caspase-3 and oxidative stress-induced apoptosis. These effects were increased by TLR4 over-expression and decreased by TLR3 over-expression. We conclude that exposure to TiO_2_ NPs causes oxidative stress, with increased H_2_O_2_ and ^**·**^OH levels leading to DNA damage and p53 activation, and induces apoptosis by releasing cytochrome c into the cytoplasm and activating caspase-3. Over-expression of TLR3 protects against oxidative stress-induced damage in response to TiO_2_ NP exposure, but over-production of TLR4 enhances the oxidative stress mediated by TiO_2_ NPs. TiO_2_ NPs induce the expression of 17 DNA damage marker genes, especially the ATM and IP6K3 genes. This indicates that the type of DNA damage caused in HepG2 cells is double strand breaks, as well as chromatin condensation, nuclear fragmentation, and apoptosis.

## Materials and methods

### Cells and cell culture

HepG2 cells were cultured in Dulbecco’s Modified Eagle’s Medium (DMEM, Nacalai Tesque, Inc., Kyoto, Japan) supplemented with 10% fetal bovine serum (FBS, Biowest, Nuaillé, France), 100 U/mL penicillin, and 100 μg/mL streptomycin (Nacalai Tesque, Inc.) at 37°C in a humidified atmosphere containing 5% CO_2_.

### Plasmids employed

TLR-encoding genes were purchased from InvivoGen (San Diego, CA, USA). The pUNO1-mcs expression vector was used as an “empty” control vector. Since pUNO1-mcs does not contain a therapeutic gene, it can be used in conjunction with other vectors of the pUNO1 family to serve as an experimental control. Overproduction of TLR3 and TLR4 was provided by transfection with pUNO-hTLR3 (which encodes the human TLR3 protein) and pUNO1-hTLR04a (CD284a) (which harbors the human TLR04a (CD284a) encoding open reading frame), respectively. HepG2 cells were seeded in 6-well plates. After overnight incubation, the cells were co-transfected with TLR3 or TLR4 expression vectors and control plasmid (pUNO1-mcs) using Lipofectamine™ LTX Reagent (Invitrogen, Carlsbad, CA, USA) according to the supplier’s protocol. Transfection efficiency of at least 50% was obtained.

### Preparation and exposure to TiO_2_ NPs

The preparation and characterization of TiO_2_ NPs were described in previous studies [26]. Briefly, nano-TiO_2_ (AeroxideR P25; Sigma-Aldrich, St Louis, MO, USA) was dispersed in distilled water and autoclaved at 120°C for 20 min. The suspension was cooled to room temperature and then sonicated for 10 min at 200 kHz using a high-frequency ultrasonic sonicator (MidSonic 600, Kaijo Corp., Tokyo, Japan). The resulting nano-TiO_2_ suspension was designated “TiO_2_ NPs”. The concentration of TiO_2_ NPs was determined using a UV–vis spectrophotometer at 370 nm (UV-1600, Shimadzu, Kyoto, Japan). The suspension was adjusted to the desired concentration by the addition of distilled water and stored at 4°C until use. The particle size distribution was measured by dynamic light scattering (Zetasizer Nano-ZS, Malvern Instruments, Malvern, UK). The aggregated particle size of the TiO_2_ NPs was determined to be 216 ± 70 nm. The size of the aggregated TiO_2_ NPs remained stable for several weeks under the indicated storage conditions. Prior to addition to the cell cultures, the suspension of TiO_2_ NPs was diluted with supplemented medium and used as described above. For the reporter gene (transfected cell) assays, the culture medium was replaced (1 day after transfection) with medium containing the TiO_2_ NPs at the indicated concentration. Specifically, TiO_2_ NPs were added to the culture medium immediately before the medium was applied to the cells. After 48 h, the cells were harvested and assayed.

### DCF assay for oxidative stress determination

The accumulation of intracellular free radicals was quantified using a ROS assay kit (OxiSelect, Cell Biolabs, Inc., San Diego, CA, USA), which employs the cell-permeable fluorogenic probe 2′,7′-dichlorodihydrofluorescein diacetate (DCFH-DA). DCFH-DA can cross cell membranes and be deacetylated by intracellular esterases to non-fluorescent 2′, 7′-dichlorodihydrofluorescein (DCFH). In the presence of ROS, DCFH is rapidly oxidized to the highly fluorescent DCF, which is readily detectable. The fluorescence intensity is proportional to the ROS levels in the cell cytosol. HepG2 cells were cultured in 96-well black plates; after overnight incubation, the cells were co-transfected with TLR3, TLR4 or control plasmid (pUNO1-mcs). After 24 h, the cells were exposed to TiO_2_ NPs for 48h, and were then incubated with DCHF-DA for 30 min in the dark. Parallel sets of wells containing freshly cultured cells, which were not treated with NPs or plasmids, and were suspended in the same concentration ratio of DPBS and DMEM, were regarded as negative controls. The fluorescence emission of DCF was monitored at regular intervals at an excitation wavelength of 480 nm and an emission wavelength of 530 nm using a fluorescence plate reader (Twinkle LB 970 Microplate Fluorometer, BERTHOLD TECHNOLOGIES GmbH & Co. KG, Calmbacher, Bad Wildbad Germany). The amount of DCF formed was calculated from a calibration curve constructed using an authentic DCF standard.

### Measurement of H_2_O_2_

The levels of hydrogen peroxide (H_2_O_2_) were measured using a hydrogen peroxide assay kit (ab102500, Abcam, Tokyo, Japan). In the presence of horseradish peroxidase (HRP), the OxiRed Probe reacts with H_2_O_2_ to produce a colored product. Following the experiment, the cells were collected in H_2_O_2_ assay buffer and then centrifuged for 15 min at 1000 × g. A total of 50 μl of the supernatant was mixed with 50 μl of the reaction mix (assay buffer: 46 μl; OxiRed Probe: 2 μl; HRP: 2 μl) and then incubated at room temperature for 10 min. The optical density at 570 nm was read with a microplate reader (Benckmark Plus microplate spectrophotometer, BioRad, [city?] CA, USA), and the H_2_O_2_ concentration was calculated according to a standard concentration curve.

### Measurement of GPX

Glutathione peroxidase activity was measured using a glutathione peroxidase assay kit provided by Cayman Chemical Company (Ann Arbor, MI, USA). Cells were washed in phosphate buffer, pH 7.4, collected by centrifugation (2000 × g for 10 min at 4°C), then homogenized in cold assay buffer (50 mM Tris-HCL, pH 7.5, 5 mM EDTA, 1 mM DTT). Following centrifugation at 10,000 × g for 15 min at 4°C, the supernatant was removed for assay. Sample (20 μl of supernatant) was added to the desired well of a 96-well plate, then 100 μL of assay buffer and 50 μl of co-substrate mixture was added. The reaction was initiated by adding 20 μl of cumene hydroperoxide to each reaction well, then mixed by shaking for second. The absorbance was read at 340 nm using a plate reader. At least 5 time points were obtained.

### Measurement of GSH

The total glutathione concentration (reduced and oxidized forms) was determined in a microtitre plate assay using a glutathione assay kit (Sigma-Aldrich). After TLR transfection and nanoparticle exposure, HepG2 cells were washed twice with phosphate-buffered saline (PBS), resuspended in a 5% 5-sulfosalicylic acid solution, then centrifuged at 10,000 × g for 10 min. Supernatant (10 μl) was mixed with 150 μl of working solution, incubated for 5 min at room temperature, then 50 μl of the diluted NADPH solution was added. The absorbance of each sample was measured at 412 nm using the plate reader, as was the absorbance of the reagent blank (10 μl of 5% 5-sulfosalicylic acid); the absorbance of the blank was then subtracted from the absorbance of each sample. The final concentration of the components in the reaction mixture was 95 mM potassium phosphate buffer, pH 7.0, containing 0.95 mM ethylenediamine tetra-acetic acid (EDTA), 0.038 mg/ml (48 μM) NADPH, 0.031 mg/ml DTNB, 0.115 units/ml glutathione reductase, and 0.24% 5-sulfosalicylic acid. All measurements were performed in triplicate; the concentration (nmoles) of GSH in the samples was calculated.

### Measurement of caspase-3 activity

All reagents for assessing caspase-3 activity were provided in a caspase-3 colormetric assay kit, (Sigma Aldrich). HepG2 cells (1 × 10^6^) were cultured in 6-well plates and treated as described above. At the end of the experiment, the cells were washed and lysed in 100 μl of lysis buffer provide in the kit, then 80 μl of the sample was added to 10 μl of the 10× assay buffer and 10 μl of in a well of a 96-well plate. The reaction mix was incubated for 10 hours at 37°C, then the absorbance was read at 405 nm.

### Gene expression analysis: PCR array

For polymerase chain reaction (PCR) array analysis, HepG2 cells (at 6 × 10^5^ cells/ml) with or without TLR4 transfection were seeded in a culture dish containing culture medium with or without TiO_2_ NPs (suspended at 10 μg/ml). After 48 h exposure to the TiO_2_ NPs, the cells were detached by mechanical dissociation and total cellular RNA was extracted using an RNeasy kit (Qiagen, MD, USA). An aliquot (1 μg) of the extracted total RNA was reverse transcribed into cDNA with random hexamer primers using a RT^2^ First Strand kit (SABiosciences/Quiagen MD, USA) and the expression of 89 human DNA damage-related genes involved in signaling pathways were examined using a RT^2^ Profiler PCR array kit (SABiosciences/Quiagen) according to the manufacturer’s instructions. PCR array analysis was performed using an ABI PRISM 7000 sequence detection system (Applied Biosystems, Singapore).

### Real-Time (RT) PCR

For mRNA expression analysis, 6 × 10^5^ HepG2 cells/ml were seeded in cell culture dishes, the cells were transfected with TLR4 expression vector and exposed to a suspension of TiO_2_ NPs at a final concentration of 10 μg/ml for 48 h, then the cells were detached and subjected to gene expression analysis. The expression of marker genes was determined using quantitative real-time PCR (RT-PCR) as follows. Total RNA and cDNA were synthesized as described for the PCR array. The PCR primers for human APEX1, ATM, GADD45A, IP6K3, MBD4, SMC1A were purchased from SABiosciences/Qiagen. The data were normalized using the housekeeping gene glyceraldehyde-3-phosphate dehydrogenase (GAPDH) as an endogenous control in the same reaction as the gene of interest [42]. The reaction mixture was composed of 12.5 μl of RT^2^ SYBR Green qPCR Master Mix (SABiosciences;/Qiagen), 1 μl of 10 μM gene-specific RT^2^ qPCR forward and reverse primers, 2 μl of cDNA, and nuclease-free water to a final volume of 25 μl. The thermocycling conditions were 95°C for 10 min, followed by 40 cycles of 95°C for 15 s and 60°C for 1 min.

### Confocal microscopy observation

Confocal laser scanning microscopy was performed using a Zeiss LSM510 microscope (Carl Zeiss, Oberkochen, Germany). HepG2 cells were cultured on cover-slips (13 mm diameter; Matsunami Glass Ind., Ltd., Osaka, Japan). The following day, cultures were transfected (using the Lipofectamine™ LTX Reagent, as described above) with the expression vector encoding TLR4. At 24 h after transfection, the culture medium was replaced with medium containing 10 μg/ml TiO_2_ NPs. Untransfected cells and cells without NP exposure were used as controls. After 48 h incubation, the cells were washed with PBS and fixed with 4% paraformaldehyde for 5 min. Fixed cells were then stained for nuclei using 1 μg/ml Hoechst33342 (Dojin Chemical**,** Japan) for 30 min in a 5% CO_2_ environment. Figures were created using NIH ImageJ software.

### Statistical analysis

Data were expressed as the mean ± SD, (*n* ≥3). All experiments were carried out independently. The data were analyzed using Student’s *t* test to evaluate the significance of differences between the treated groups and control groups. Statistical significance was accepted at *P* < 0.05.

## Abbreviations

TiO_2_ NPs: Titanium dioxide nanoparticles
TLRs: Toll-like receptors
PCR: Polymerase chain reaction
ROS: Reactive oxygen species
DCF: Dichlorofluoroscein
NADH: Nicotinamide adenine dinucleotide hydrogen
APEX1: Apurinic/Apyrimidinic exonuclease 1
ATM: Ataxia telangiectasia mutated
GADD45A: Growth arrest and DNA-damage-inducible, alpha
IP6K3: Inositol hexakisphosphate kinase 3
MBD4: Methyl-CpG binding domain protein 4
SMC1A: Structural maintenance of chromosomes
H_2_O_2_: Hydrogen peroxide
GPx: Glutathione peroxidase
GSH: Reduced glutathione
RAD: Radiation associated damage “DNA repair gene”
